# Pediatric supraglottic airway devices in clinical practice: A prospective observational study

**DOI:** 10.1186/s12871-017-0403-6

**Published:** 2017-09-02

**Authors:** Maren Kleine-Brueggeney, Anne Gottfried, Sabine Nabecker, Robert Greif, Malte Book, Lorenz Theiler

**Affiliations:** 1 Department of Anaesthesiology and Pain Therapy, Inselspital, University Hospital Bern, University of Bern, Freiburgstrasse, 3010 Bern, Switzerland; 2 Present address: Department of Anaesthesia, Evelina London Children’s Hospital, Guy’s and St. Thomas’ NHS Foundation Trust, London, UK; 3 Present address: Department of Anaesthesia, Klinikum Oldenburg AöR, 26133 Oldenburg, Germany

**Keywords:** Airway management, Laryngeal mask airway, Supraglottic airway device, Pediatric anesthesia, General anesthesia

## Abstract

**Background:**

Supraglottic airway devices (SGA) are commonly used in pediatric anesthesia and serve as primary or back-up devices for difficult airway management. Most SGA are marketed without proper clinical evaluation. The purpose of this study was to evaluate the performance of the pediatric LMA Supreme™, Air-Q® and Ambu® Aura-i™.

**Methods:**

This prospective observational study was performed at Bern University Hospital, Switzerland. With ethics committee approval and a waiver for written informed consent 240 children undergoing elective surgery with an ASA class I-III and a weight of 5-30 kg were included. Three different pediatric supraglottic airway devices were assessed: The LMA Supreme™, Air-Q® and Ambu® Aura-i™***.*** Primary outcome parameter was airway leak pressure. Secondary outcome parameters included first attempt and overall success rate, insertion time, fiberoptic view through the SGA, and adverse events. The primary hypothesis was that the mean airway leak pressure of each tested SGA was 20 cmH_2_O ± 10%.

**Results:**

None of the SGA showed a mean airway leak pressure of 20 cmH_2_O ± 10%, but mean airway leak pressures differed significantly between devices [LMA Supreme™ 18.0 (3.4) cmH_2_O, Air-Q® 15.9 (3.2) cmH_2_O, Ambu® Aura-i™ 17.3 (3.7) cmH_2_O, *p* < 0.001]. First attempt success rates (LMA Supreme™ 100%, Air-Q® 90%, Ambu® Aura-i™ 91%, *p* = 0.02) and overall success rates (LMA Supreme™ 100%, Air-Q® 91%, Ambu® Aura-i™ 95%, *p* = 0.02) also differed significantly. Insertion times ranged from 20 (7) seconds (Air-Q®) to 24 (6) seconds (LMA Supreme™, <*p* = 0.005). Insertion was rated easiest with the LMA Supreme™ (very easy in 97% vs. Air-Q® 70%, Ambu® Aura-i™ 72%, *p* < 0.001). Fiberoptic view was similar between the SGA. Adverse events were rare.

**Conclusions:**

Airway leak pressures ranged from 16 to 18 cmH_2_O, enabling positive pressure ventilation with all successful SGA. The highest success rates were achieved by the LMA Supreme™, which was also rated easiest to insert.

**Trials Registration:**

ClinicalTrials.gov, identifier NCT01625858. Registered 31 May 2012.

## Background

Pediatric supraglottic airway devices (SGA) are being used with great success and over 50% of general anesthetic procedures are managed with SGA [1]. A review article showed that compared to tracheal intubation, the use of SGA results in a decreased number of postoperative airway complications like desaturation, laryngospasm, coughing or breath holding [2]. Also, a reduction in postoperative nausea and vomiting [3, 4] and faster recovery [4] has been reported. More advanced second generation pediatric SGA have been released, but often without evaluation in comparative, industry-independent studies prior to marketing. This is reflected by a survey of the Association of Paediatric Anaesthetists of Great Britain and Ireland in which 77% stated that trials assessing pediatric SGA were necessary [1]. Interestingly, 88% preferred first generation pediatric SGA over second generation SGA [1]. Clinical trials on pediatric SGA are scarce [5–7]. Many devices have been tested in adults [8, 9], but those results cannot be extrapolated to children.

This prospective observational study evaluates the performance of three pediatric SGA in 240 children: The pediatric LMA Supreme™ (LMA-S™, LMA Company, LeRocher, Victoria, Mahe, Seychelles), AirQ® (Cookgas® LLC, St. Louis, USA) and Ambu® Aura-i™ (Aura-i™, Ambu A/S, Ballerup, Denmark). Based on our previous study [10] and published literature [5], pediatric SGA are expected to provide airway leak pressures of about 20cmH_2_O. Our primary hypothesis was that the mean airway leak pressure of each tested SGA was 20 cmH_2_O ± 10%.

## Methods

This prospective observational study evaluated the clinical performance of three pediatric SGA. It was performed at the Department of Anaesthesiology and Pain Therapy at the Inselspital, Bern University Hospital, Switzerland. Ethical approval was given by the Internal Review Board of the Bern University Hospital. The internal review board issued a waiver of written informed consent. The study was registered through the international trials registry ClinicalTrials.gov under the identifier NCT01625858.

### Participants, inclusion and exclusion criteria

Data of 240 patients were prospectively obtained. Patients were boys and girls, 0–17 years old, weighing 5-30 kg, with an ASA physical status I-III, and scheduled for elective surgery under general anesthesia with an SGA and a planned operation time < 4 h. Exclusion criteria were risk of aspiration, body mass index >35 kg m^−2^, cervical spine disease, congenital anatomical malformation, known difficult airway, upper respiratory tract symptoms within 10 days, preoperative sore throat or poor dentition.

### Devices

**Fig. 1:**
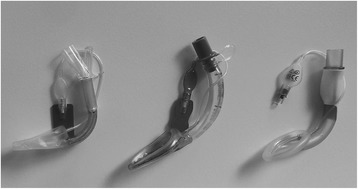
The three different supraglottic airway devices evaluated in this study. From left to right: Size 2 of the LMA-S^™^, Air-Q® and the Aura-i^™^

Three pediatric SGA were compared: The LMA-S^™^ and the two intubation SGA Air-Q® and Aura-i™ (Fig. 1). The LMA-S™ comes with a gastric channel, allowing for a functional separation of the digestive and respiratory tract. The AirQ® and the Aura-i™ do not feature a gastric channel, but are SGA designed for management of difficult airways and for intubation via the SGA. The SGA were used according to manufacturer’s recommendations and the size was determined by patients’ weight. To avoid selection bias, the specific SGA investigated was not chosen by the anesthesiologist in charge, but, depending on availability on stock, by a study nurse who was not involved in the anesthetic management of the child.

### Anesthesia

Anesthesia followed the standard operating procedures of the Children’s Hospital Bern and was standardized to guarantee equal depth of anesthesia. After premedication and preoxygenation, anesthesia was induced by inhalation of nitrous oxide and sevoflurane with final end-tidal sevoflurane concentrations of 6%, or intravenously with propofol (4 mg kg^−1^) and fentanyl (1–3 μg kg^−1^) and/or alfentanil (20 μg kg^−1^). Adequate depth of anesthesia was verified by loss of eyelash reflex, symmetric small pupils, and lack of physical reaction to jaw-thrust [10]. Bag mask ventilation was provided and the SGA was lubricated with water-based K-Y Lubricating Jelly (Johnson & Johnson Medical Limited, Gargrave, Skipton, United Kingdom). An assistant performed the jaw-thrust manoeuvre. The SGA was then inserted by an attending, a resident or by a certified anesthesia nurse, supervised by one of the senior pediatric anesthesiologists. A maximum of three insertion attempts was performed and cuff pressure was set to 60 cmH_2_O using a manometer (VBM GmbH, Sulz, Germany or Rüsch GmbH, Kernen, Germany). SGA were secured with an adhesive tape around the SGA spanning both cheeks. All patients were ventilated using pressure controlled ventilation. Following clinical standards, the SGA were usually removed in a deeply sedated, spontaneously breathing patient. The time of removal ultimately depended on the decision of the anesthesiologist.

### Measurements

All data were recorded by a member of the research team who was not involved in clinical care. Recorded demographic and anesthesia-related data included sex, age, weight, height, ASA class, type of anesthesia induction, and duration of anesthesia.

Primary outcome was defined as mean airway leak pressure [cmH_2_O] after placement of the SGA [10–12]: Cuff pressure was set to 60cmH_2_O. Gas flow was 3 l min^−1^ and the expiratory valve was closed at 30cmH_2_O. The airway pressure at which a steady state was reached was determined.

Secondary outcome parameters were first attempt and overall success rate. Failures and reasons for device failure were noted. Insertion time was measured from face mask removal to successful ventilation of the lungs [13, 14]. Subjective difficulty of handling was evaluated by the anesthesiologist on a scale from 1 to 5 (1: very easy, 2: easy, 3: difficult, 4: very difficult, 5: impossible), as done before [8, 15, 16]. Feasibility of gastric catheter insertion was noted.

When deemed clinically desirable by the attending anesthesiologist, a fiberscope was inserted through the airway port and in these cases, fiberoptic view was graded as full view of the glottis (1), partial view of the glottis (2), only epiglottic structures seen (3), or no glottic/epiglottic structures visible (4), as done before [17–19]. Epiglottic downfolding was noted.

Airway interventions after initial successful ventilation were noted as well as adverse events like aspiration, regurgitation, bronchospasm, obstruction, dental, tongue or lip trauma, and blood on the removed SGA. The day after surgery patients and/or parents were interviewed about side effects like sore throat, hoarseness, dysphagia, tongue numbness, and postoperative nausea and vomiting (all graded as no/mild/moderate/severe).

### Statistical analysis

Based on earlier studies, we expected leak pressures for pediatric second generation supraglottic airway devices to be around 20cmH_2_O [5, 10]. Our primary hypothesis was that the mean airway leak pressure of each tested SGA was 20 cmH_2_O ± 10%. More precisely this means that the 95% confidence interval (CI) for mean airway leak pressure of each tested SGA is within 20 cmH_2_O ± 10% (18–22 cmH_2_O). Given these expectations, a sample size calculation with a two-sided alpha level of 0.05 and a power of 0.9 calculated 63 patients per cohort necessary. To compensate for drop outs or missing data, we planned to include 80 patients per cohort.

Binary data were analysed by Chi square or by Fisher’s exact test if more than 20% of expected values were below 5. Ordinal data were evaluated using Kruskal-Wallis test. Normal distribution for continuous data was tested using Q-Q plots and Shapiro-Wilk W test. Independent samples Kruskal-Wallis test was used for analysis of non-parametric data and ANOVA for analysis of parametric data. For multiple comparisons of statistically significant data, Student’s t-test was used for parametric data and Mann–Whitney u-test for non-parametric data. Bonferroni correction was applied. We performed a subgroup analysis with subgroups according to children’s weight (subgroup 1: 5–9.9 kg; subgroup 2: 10–19.9 kg; subgroup 3: 20-30 kg).

Data are presented as numbers (%) for binary and ordinal data, as mean (SD) for parametric data, or as median (IQR) for nonparametric data. A probability of *p* < 0.05 was considered statistically significant. Data were analysed using Stata V.13.1 (StataCorp, College Station, TX, USA).

## Results

Data of 240 SGA insertions were evaluated. 201 children (84%) were male and 39 (16%) female. Distribution of gender and other demographic data did not differ between groups (*p* > 0.05, Table 1). Age ranged from 0.5 to 12.9 years. Weight ranged from 5.6 to 30.0 kg.

**Table 1:** Patient characteristics and data regarding anesthesia

	LMA-S^™^	Air-Q®	Aura-i^™^	*p*-value
	*n* = 80	*n* = 80	*n* = 80	
Male sex - number (%)^a^	64 (80)	66 (83)	71 (89)	0.30
Age – years, median (IQR)^b^	5.0 (3.0–7.8)	5.0 (2.4–6.6)	5.0 (2.6–6.6)	0.47
Weight – kg, median (IQR)^b^	19.2 (15.0–25.8)	19.3 (13.0–24.0)	19.0 (13.3–23.0)	0.57
Height – cm, median (IQR)^b^	115 (97–127)	115 (90–123)	112 (97–120)	0.38
ASA I/II/III, number (%)^c^	55/24/1 (69/30/1)	63/16/1 (79/20/1)	60/19/1 (75/24/1)	0.64
Induction: inhalational/intravenous, number (%)^a^	39/41 (49/51)	30/50 (38/63)	29/51 (36/64)	0.21
Duration of anesthesia – min, median (IQR)^b^	100 (80–126)	87 (73–114)	93 (77–121)	0.09

^a^test statistics: Chi square test;

^b^test statistics: Kruskal Wallis test;

^c^test statistics: Fisher’s exact test

### Primary outcome

For none of the SGA did the 95% confidence interval of airway leak pressure range between 18 and 22 cmH_2_O, leading to rejection of the hypothesis for all three SGA. Mean airway leak pressures differed significantly between groups (*p* < 0.001) and were 18.0 (3.4) cmH_2_O with the LMA-S™, 15.9 (3.2) cmH_2_O with the Air-Q® and 17.3 (3.7) cmH_2_O with the Aura-i™ (Table 2). In the posthoc analysis, leak pressure with the Air-Q® was significantly lower than with the LMA-S™ or the Aura-i™.

**Table 2:** Supraglottic airway device performance. Bonferroni correction was applied for multiple comparisons

	LMA-S™	Air-Q®	Aura-i™	*p*-value
	*n* = 80	*n* = 80	*n* = 80	
First attempt success: number (%); [95% CI]^a^	80 (100); [95 to 100]^b^	72 (90); [79 to 95]	73 (91); [81 to 96]	0.02
Overall success: number (%); [95% CI]^c^	80 (100); [95 to 100]^d^	73 (91); [81 to 96]	76 (95); [87 to 99]	0.02
	*n* = 80	*n* = 73	*n* = 76	
Leak pressure - cmH_2_O: mean (SD); [95% CI]^e^	18.0 (3.4); [17.2 to 18.7]	15.9 (3.2); [15.1 to 16.6]^f^	17.3 (3.7); [16.4 to 18.1]	<0.001
Number of attempts 1/2/3: number (%)^c^	80/0/0 (100/0/0)	72/1/0 (99/1/0)	73/2/1 (96/3/1)	0.28
Ease of insertion: number (%)^g^	77/2/0/0 (97/3/0/0)^b^	51/21/1/0 (70/29/1/0)	54/15/6/0 (72/20/8/0)	<0.001
Insertion time - seconds: mean (SD); [95% CI]^e^	24 (6); [23–25]^d^	20 (7); [19–22]	22 (7); [20–24]	0.005
	*n* = 68	*n* = 45	*n* = 51	
Fiberoptic view: number (%)^h^	27/29/10/2 (40/43/15/3)	26/14/4/1 (58/31/9/2)	22/24/5/0 (43/47/10/0)	0.41
Epiglottic downfolding: number (%)^i^	11 (17)	5 (11)	5 (10)	0.51

^a^test statistics: Chi square test;

^b^statistically different to Air-Q® and Aura-i™;

^c^test statistics: Fisher exact test;

^d^statistically different to Air-Q®;

^e^test statistics: ANOVA;

^f^statistically different to LMA-S™ and Aura-i™;

^g^graded as very easy/easy/difficult/very difficult; data missing for 1 LMA-S™ and 1 Aura-i™; test statistics Fisher exact test;

^h^fiberoptic view graded as 1: full view of the glottis, 2: partial view of the glottis, 3: only epiglottic structures seen, 4: no glottic/epiglottic structures visible [17], test statistics: Fisher exact test;

^i^test statistics: Chi square test, data missing for 2 LMA-S™ and 1 Air-Q®

### Secondary outcomes

First attempt and overall success rates differed significantly between groups (*p* = 0.02, Table 2). The LMA-S™ achieved a 100% first attempt success rate. This was statistically significantly better than the first attempt success rate of the Air-Q® or the Aura-i™. The overall success rate of the LMA-S™ was also statistically significantly better than that of the Air-Q®. For the LMA-S™, the 95% confidence interval of first attempt success rate was above 90% (Table 2).

Reasons for device failures were massive air leaks (7 Air-Q®; 4 Aura-i™), but no problems with insertion of the SGA. There was no difference in the number of insertion attempts (*p* = 0.28, Table 2).

Insertion time differed significantly between groups (*p* = 0.005, Table 2). The Air-Q® was fastest to insert and this was statistically significantly different to the LMA-S™.

Ease of insertion was best with the LMA-S™ (Table 2) and this was statistically significantly different to the other two SGA.

Gastric catheterisation was possible in 99% of LMA-S™ cases. Due to the lack of a gastric tube this was not possible with the Air-Q® or the Aura-i™. Fiberoptic view was similar with all three SGA (*p* = 0.41, Table 2). A full view of the glottis was achieved in 40% to 58%, and epiglottic downfolding was rare (10–17%, Table 2).

Several SGA leaked air or dislodged after initially successful ventilation, during lateral positioning for a caudal block (0 LMA-S™, 2 Air-Q®, 2 Aura-i™, *p* = 0.40) or intraoperatively (3 LMA-S™, 4 Air-Q®, 2 Aura-i™, *p* = 0.70). Of the leaks occurring intraoperatively, all leaks with the Air-Q® could be corrected by minor airway interventions like pushing or taping down intraoperatively, while all leaks with the LMA-S™ and the Aura-i™ led to removal of the SGA.

### Adverse events and side effects

Two children regurgitated at emergence from anesthesia (Air-Q®). One child developed a laryngospasm requiring intubation and tracheal suctioning revealed some brownish secretion (Aura-i™). In all three cases, the postoperative course was absolutely uneventful with normal auscultation, continuous SpO_2_ > 95% at room air, and timely discharge. Several children (1 LMA-S™, 1 Air-Q®, 1 Aura-i™) developed a short period of slight obstruction during or after surgery. Laryngospasm developed with one Air-Q® and one Aura-i™ during surgery and with one LMA-S™ and two Aura-i™ after surgery. One child with an Aura-i™ with bronchospasm was intubated. All other spasms resolved spontaneously or by deepening the anesthesia. One child with an Air-Q® showed minor mucosal trauma. Blood stains were observed in 2 LMA-S™, 1 Air-Q® and 5 Aura-i™.

There was no difference in sore throat (3–4%, *p* = 0.76), hoarseness (3–9%, *p* = 0.37), numbness of the tongue (0–1%, *p* = 1.00), nausea (9–22%, *p* = 0.051), or vomiting (7–15%, *p* = 0.23) between groups. The only difference between groups was dysphagia (*p* = 0.01), resulting from a higher rate of dysphagia with the LMA-S™ (6%) versus the other groups (0%). This missed statistical significance in the posthoc comparison with Bonferroni correction.

### Subgroup analysis

Subgroup analysis was performed for subgroups according to weight (Table 3), as done previously [10]. Since numbers in subgroup 1 (5–9.9 kg) were low statistical analysis was performed for subgroups 2 and 3 only. Fiberoptic view significantly differed between subgroups for the LMA-S™ and for the Aura-i™, with better views in children weighing 20-30 kg compared to children weighing 10–19.9 kg. Insertion times differed for the Aura-i™ with faster insertion in children weighing 10–19.9 kg compared to children weighing 20-30 kg (Table 3).

**Table 3:** Subgroup analysis according to body weight for each device. Since numbers in subgroup 1 are low statistical analysis was performed comparing subgroup 2 and 3 only

		Group 1	Group 2	Group 3	*p*-value
		(5–9.9 kg)	(10–19.9 kg)	(20–30 kg)	
LMA-S™	Number of patients	1	39	40	
	First attempt success, n (%)^a^	1 (100)	39 (100)	40 (100)	1.00
	Overall success, n (%)^a^	1 (100)	39 (100)	40 (100)	1.00
	Leak pressure - cmH_2_O, median (IQR)^b^	16	18 (16–19)	18 (16–20)	0.73
	Insertion time - seconds, median (IQR)^b^	21	24 (21–28)	22 (19–27)	0.13
	Fiberoptic view, n (%)^c^	0/0/1/0 (0/0/100/0)	9/15/7/2 (27/45/21/6)	18/14/2/0 (53/41/6/0)	0.048
Air-Q®	Number of patients	5	36	39	
	First attempt success, n (%)^a^	5 (100)	33 (92)	34 (87)	0.71
	Overall success, n (%)^a^	5 (100)	33 (92)	35 (90)	1.00
	Leak pressure - cmH_2_O, median (IQR)^b^	20 (17–22)	16 (15–18)	15 (12–17)	0.16
	Insertion time - seconds, median (IQR)^b^	17 (14–22)	21 (17–24)	20 (15–24)	0.61
	Fiberoptic view, n (%)^c^	2/1/1/0 (50/25/25/0)	10/9/0/1 (50/45/0/5)	14/4/3/0 (67/19/14/0)	0.054
Aura-i™	Number of patients	2	41	37	
	First attempt success, n (%)^a^	2 (100)	38 (93)	33 (89)	0.70
	Overall success, n (%)^a^	2 (100)	40 (98)	34 (92)	0.34
	Leak pressure - cmH_2_O, median (IQR)^b^	13; 20	18 (15–19)	18 (15–20)	0.17
	Insertion time - seconds, median (IQR)^b^	10; 28	19 (15–23)	25 (20–30)	<0.001
	Fiberoptic view, n (%)^c^	1/0/0/0 (100/0/0/0)	5/17/5/0 (19/63/19/0)	16/7/0/0 (70/30/0/0)	<0.001
*p*-values comparing the 3 SGA	First attempt success ^d^	NA	0.21	0.05	
	Overall success ^d^	NA	0.11	0.10	
	Fiberoptic view^d^	NA	0.06	0.19	
	Leak pressure^e^	NA	0.06	<0.001	
	Insertion time^e^	NA	<0.001	0.007	

^a^test statistics Fisher exact test;

^b^for variables with *n* ≤ 3 original data are given instead of median with IQR; test statistics: Mann Whitney U test;

^c^fiberoptic view graded as 1: full view of the glottis, 2: partial view of the glottis, 3: only epiglottic structures seen, 4: no glottic/epiglottic structures visible; 17 test statistics Fisher exact test

^d^within subgroup comparison, test statistics Fisher exact test;

^e^within subgroup comparison, test statistics Kruskal Wallis test

Comparison of the three SGA showed statistically significant differences in insertion time within subgroups 2 and 3, and for leak pressure within subgroup 3 (Table 3).

## Discussion

Confidence intervals of mean airway leak pressure were below the range of 18–22 cmH_2_O with all three SGA. Leak pressures ranged from 16 to 18 cmH_2_O and differed between devices. The Air-Q® showed the lowest leak pressures, which were similar to previous studies [6, 7, 20]. A previous study on the Ambu® Aura-i™ reported mean airway leak pressures of 16 cmH_2_O [7], while this was 18 cmH_2_O in our study. Leak pressure of the LMA Supreme™ was similar to a previous study reporting leak pressures of 20 cmH_2_O [5]. Overall, leak pressures were lower than stated in our hypothesis, but still enabled positive pressure ventilation with all successful SGA and it is questionable whether the detected differences in leak pressure are of clinical relevance. Of note, our hypothesis was not based on a level of leak pressure that is associated with successful positive pressure ventilation of children, but on earlier results from other SGA [5, 10]. Taking the different studies into account, it seems that the range of measured leak pressures with different SGA is wide and starts at 16cmH_2_O. Leak pressures of SGA in children are therefore lower than those in adults [8, 21], but the clinically relevant point is that even with these low leak pressures positive pressure ventilation of children is possible.

We previously published a prospective study on the performance of the pediatric i-gel® and AuraOnce™ [10]. In that study the leak pressure of the i-gel was 22 (5) and leak pressure of the AuraOnce™ was 19 (3) cmH2O. The study used the same methods regarding anesthesia and procedures as the current study, but included children weighing 5-50 kg. Other studies of the i-gel® showed similar leak pressures [22, 23]. Interestingly, leak pressure therefore seems to be highest with the i-gel®, which does not feature an inflatable cuff.

First attempt success rates in our study were lower than in other publications, which showed rates of 100% for the Aura-i™ [7] and the Air-Q® [6, 7], and 96–97% for the LMA-S™ [5, 23]. Since SGA performance depends on children’s age and weight [10], these results might be explained by differences in demographics. In our previous study, first attempt success rates were 89% with the i-gel® and 90% with the Ambu® AuraOnce™, similar to data from the Air-Q® (90%) and Ambu® Aura-i™ (91%) in the present study. Thus, the LMA-S™ with its 100% first attempt success rate performed particularly well. Similarly, the ease of insertion, as graded by the person inserting the SGA on a commonly used subjective scale [9, 17, 18], was best with the LMA-S™.

We previously defined a first attempt success rate with a 95% confidence interval above 90% as the minimal desirable target for airway management devices [24]. To our knowledge, no other definition of a minimal threshold for airway management devices exists. Several studies have shown success rates of ≥95% [5–7, 25], and we therefore postulated that a 95% confidence interval above 90% should be a realistic, achievable goal for airway management devices. In the present study, only the LMA-S™ achieved this target (95% CI of first attempt success rate 95–100%).

Fiberoptic view was similar between the three SGA in the present study, but a full view of the vocal cords was achieved in only 40–58%. In contrast, our previous study showed much better fiberoptic views with the i-gel® and the AuraOnce™ (full view in 88 and 89%, respectively) [10]. The full view of the glottic opening from the orifice of the SGA is likely to reflect alignment of the SGA and the larynx and could play an important role for intubation through SGA. Interestingly, the Air-Q® and Aura-i™, but not the i-gel® and AuraOnce™, are specifically marketed for intubation. Success rates of 95–100% have been described for fiberoptically guided intubation through the Air-Q® [7, 26]. However, we showed that without fiberoptic guidance, intubation success rates through the pediatric Air-Q® and Aura-i™ are low, which is in line with the results of the fiberoptic assessment [27].

Air-Q® insertion was fastest in the present study. However, the faster insertion will most likely be of minimal clinical relevance.

In three children, anesthesia personnel reported regurgitation or possible aspiration. Clinical evaluation of minor aspiration remains difficult and it is unclear whether these children aspirated or not. In any case, their clinical course was completely uneventful. Temporary airway obstruction occurred in several children and rates were similar to reported rates [25]. Most airway obstructions occurred towards the end of anesthesia. Due to the observational character of the study, the time of removal of the SGA varied and it is unclear how the depth of anesthesia influenced the occurrence of airway obstructions. Other adverse events like trauma or side effects such as sore throat were minor and rare.

More boys than girls were included in this study. This was due to a high percentage of circumcisions and likely does not influence outcomes. The ratio of boys and girls did not differ between groups.

The study was not randomized but observational, carrying the risk of selection bias. To prevent that anesthesiologists could choose a device of their liking, a study nurse who was not involved in anesthetic management of the patient chose the device. Also, demographic data were equally distributed between groups, showing that selection bias is rather unlikely.

Finally, our study included few patients weighing less than 10 kg (*n* = 8). SGA tend to perform differently in smaller and larger children [7, 10] and our results have to be interpreted with caution when transferred to younger patients. However, there was no statistical difference in age between the different SGA groups.

## Conclusions

Leak pressure was lowest with the Air-Q®, but with leak pressures ranging from 16 to 18cmH_2_O, positive pressure ventilation was possible with all successfully inserted SGA. The LMA-S™ reached the highest first attempt and overall success rates, and was particularly easy to insert.

## Abbreviations

ASA: American Society of Anesthesiologists
LMA-S: Laryngeal Mask Airway Supreme
SGA: Supraglottic airway device
